# A scoping review of COVID-19 online mis/disinformation in Black communities

**DOI:** 10.7189/jogh.12.05026

**Published:** 2022-07-23

**Authors:** Janet Kemei, Dominic A Alaazi, Mia Tulli, Megan Kennedy, Modupe Tunde-Byass, Paul Bailey, Ato Sekyi-Otu, Sharon Murdoch, Habiba Mohamud, Jeanne Lehman, Bukola Salami

**Affiliations:** 1 University of Alberta, Faculty of Nursing, Edmonton, Alberta, Canada; 2 Black Physicians of Ontario, University of Ontario, Department of Obstetrics and Gynecology, Toronto, Ontario, Canada; 3 Black Health Alliance, Toronto, Ontario, Canada; 4 Black Opportunity Fund, Toronto, Ontario, Canada; 5 Health Association of African Canadians, Cherry Brook, Nova Scotia, Canada; 6 IMPACT Institute of Canada, Edmonton, Alberta, Canada; 7 Black Canadian Women in Action, Edmonton, Alberta, Canada

## Abstract

**Background:**

Mis/disinformation has reached an epidemic level with the COVID-19 virus and can be largely attributed to the growing digitalization of information and its rapid transmission via social media. Approximately 96% of Canadians and 80% of Americans report encountering COVID-19 dis/misinformation on at least one social media site/app. COVID-19 dis/misinformation promotes scepticism and a lack of confidence in COVID-19 interventions. Black people have been disproportionately affected by the COVID-19 pandemic in terms of negative impacts on their livelihoods and are also more likely to be hesitant to receive the COVID-19 vaccine. Dis/misinformation contributes to high rates of COVID-19 infection and low uptake of COVID-19 vaccination. Hence, the purpose of this scoping review was to map out the nature and extent of current research on COVID-19 disinformation among Blacks in Africa and the African diaspora.

**Methods:**

We searched and reviewed articles from major databases such as MEDLINE, EMBASE, and CINAHL. Our search strategy involved the following concepts: 1) COVID-19, including variants; 2) misinformation, conspiracy theories, and fake news, and modes of misinformation transmission such as social media; and 3) Blacks or people of African descent, or the African diaspora. We retrieved 600 articles that were independently screened by two researchers. We included studies focusing on 1) Black people living inside or outside Africa; and 2) COVID-19 online dis/misinformation among this population. A total of 19 studies fit our inclusion criteria. We used a thematic analysis to analyse qualitative data.

**Results:**

Our findings indicate Black people are accessing and often sharing online disinformation and misinformation primarily through social media platforms such as WhatsApp, Facebook, Twitter, YouTube, and Instagram. Dis/misinformation concerns the origin of COVID-19, transmission, prevention, and treatment of COVID-19, assertions of race immunity to the virus, distrust in government and health organizations, and intervention research and programming.

**Conclusions:**

There is a global paucity of literature addressing COVID-19 online dis/misinformation among Black people. Dis/misinformation can fuel vaccine hesitancy and threaten the goal of herd immunity. Knowledge of the impact and implications of COVID-19 online dis/misinformation is necessary to inform public health interventions in Black communities.

COVID-19 online information is characterized by a copious blend of facts, fallacies, rumours, and speculations. The World Health Organization (WHO) thus warns of the dangers of a COVID-19 infodemic containing both accurate and inaccurate information about the pandemic. Spreading digitally and through person-to-person communication, the infodemic prevents accurate public health information from reaching its intended audience [1]. The COVID-19 infodemic features content that is inadvertently misleading with no intention of hurting others (misinformation) and another that is spread with an intent to cause harm (disinformation) [1-3]. The WHO therefore views COVID-19 disinformation as scientifically inaccurate claims disseminated with an intent to deceive the public and undermine public health response to the pandemic. In more sophisticated forms of disinformation, the fabricated claims are purposely entangled with semi-accurate information and repackaged as 'alternative facts' to enhance the former's appeal, authenticity, and believability [3].

COVID-19 disinformation is alarmist by design, and the messaging is typically purposed to trigger fear and cause people to think and act contrary to stipulated public health guidelines while drawing on verifiable scientific but incomplete information from government and public health institutions to buttress such agendas [1]. Hence, it could be difficult to distinguish between misinformation and disinformation and their respective effects on increasing personal and public health risks as well as people’s confidence in the COVID-19 vaccines. The differentiation between disinformation and misinformation is further complicated by the difficulty of determining the intent of those who peddle falsehood about COVID-19. Thus, the COVID-19 infodemic can best be conceptualized in terms of the sheer amount of incorrect information in the public domain. Data from recent surveys demonstrate that 80% of American and 96% of Canadian online news consumers have been exposed to at least one form of incorrect COVID-19 information. African fact-checking agencies refuted more than 1300 similarly misleading reports as of March 2021 [4-6].

While pandemic mis/disinformation is not atypical, the phenomenon has reached an epidemic level with the COVID-19 virus and can be largely attributed to the growing digitalization of information and its rapid transmission via social media [7]. The availability of social media platforms (eg, Twitter, Facebook, Instagram, YouTube, TikTok, etc.) and other online resources have aided the growth and globalisation of COVID-19 mis/disinformation. Combined with modern communication technologies such as smartphones and computers, these sources permit inexpensive production, dissemination, and consumption of fake news. As such, concocted information about COVID-19 has eclipsed reliable information from credible sources (eg, WHO, local health authorities, and Centers for Disease Control) in terms of its global reach, spreading more rapidly and reaching millions of consumers at a record speed [8,9].

The widespread imposition of lockdowns and stay-at-home orders as containment strategies increased people's dependence on social media and other online sources, not only for information about the pandemic itself but also as (virtual) places for social interaction, emotional connections, and entertainment [10], thereby contributing inadvertently to the growth of the COVID-19 infodemic. Indeed, public health directives from national authorities enforcing social distance and limiting population mobility have resulted in an exponential increase in social media use, both in terms of user numbers and time spent on these messaging platforms [11]. For example, Facebook recorded up to a 70% increase in the amount of time spent on its instant messaging platforms since the start of the pandemic [12]. Given the dynamic nature and rapid evolution of the COVID-19 pandemic, this dependence increasingly exposes users to inaccurate and malicious information about COVID-19 and the vaccines developed to curtail its spread.

The motives underpinning COVID-19 disinformation vary considerably but typically include economic and political interests. By sensationalizing the pandemic through the creation and dissemination of viral content, the creators of such disinformation can redirect online traffic to private websites, where they generate income from product advertisement and the sale of illicit substances (eg, alcoholic beverages) that are marketed as COVID-19 therapies, immune boosters, and anti-COVID-19 remedies [13]. Other individual-level factors, such as one's need for self-promotion or reaffirmation of a sense of competence, could also drive the production and dissemination of COVID-19 disinformation [14].

Some individuals may also use disinformation as a tool to discredit political opponents and promote certain ideologies [15]. Politicians and political parties may push for state-sponsored disinformation in order to promote or even legitimize certain political agendas. For example, in the earlier stages of the pandemic, the Chinese and US governments engaged in accusations and counteraccusations about its origins, while others deliberately misreported its fatalities [16]. In some instances, COVID-19 disinformation has been tied to and conflated with broader notions of citizenship, democratic freedoms, and rights to self-determination, garnering support for public resistance against recommended preventive measures such as mandatory quarantine and mass vaccination [17].

The social and public health impact of COVID-19 disinformation can be devastating. It notably breeds distrust for state institutions, the science community, and health care workers and may thus help derail efforts to contain the rapid transmission of the virus. Disinformation engenders “risky citizen behaviour” and disregard for safety protocols, including anti-mask and anti-vaccine rhetoric, and even protests undermining public health messaging [3]. Beyond the threats to public health, COVID-19 disinformation has inspired discrimination against and hatred for minority groups perceived as being the originators of the pandemic, as well as the destruction of public infrastructure (eg, 5G telecommunication systems) perceived as aiding the transmission of the virus [8].

The public health impacts of COVID-19 disinformation are comparatively more apparent in Black communities, where infection rates are among the highest and vaccine uptake remains well below national averages [18]. COVID-19 disinformation and scepticism in black communities are partly fueled by previously unfortunate public health work, including “Generations of experimentation on Black Americans” that have left long-lasting psychological scars and a general distrust of public health systems [18].

Landmark medical experiments, such as the Tuskegee Syphilis Study that denied treatment for Black men and the Henrietta Lacks scandal involving unconsented extraction of cancer cells, have been invoked to validate COVID-19 disinformation in Black communities [19,20]. In northern Nigeria, 11 children died, and several others were paralysed in a Pfizer meningitis drug trial in the 1990s; although this matter was eventually settled out-of-court [21], it contributed to a culture of mistrust of public health interventions in African countries. The contemporary everyday experience of medical racism, including difficulty accessing health insurance and general mistreatment of Black patients at the hands of non-Black health workers, exacerbates the predisposition of Black communities to COVID-19 disinformation [19].

Accordingly, COVID-19 disinformation in specific ethno-racial communities has attracted much scholarly interest [22,23]. Empirical studies investigating COVID-19 disinformation in Black communities have included surveys and expert observational studies. However, given the novelty of the pandemic, these studies are understandably sporadic, localized, and inconclusive in terms of their findings on COVID-19 online disinformation in Black communities. Therefore, the purpose of this scoping review was to map out the nature and extent of current research on COVID-19 disinformation among Blacks in Africa and the African diaspora. The review addresses three specific objectives: 1) to synthesize current research and identify instances of COVID-19 online disinformation among Black populations in Africa and the African diaspora, 2) to identify measures to address COVID-19 disinformation in Black communities, and 3) to identify gaps for future research on pandemic disinformation among Blacks.

The review presents Black people’s perception of COVID-19 as shared on social media and other online platforms, providing insights for understanding and addressing COVID-19 disinformation in Black communities.

## METHODS

According to Davis and Gould [24], a scoping review “involves the synthesis and analysis of a wide range of research and non-research material to provide greater conceptual clarity about a specific topic or field of evidence”. To generate evidence on COVID-19 online misinformation among Black populations and strategies to tackle it based on available international evidence, we conducted a scoping review guided by Levac’s scoping review methodology [25]. We reviewed articles that examined COVID-19 online disinformation/misinformation.

**Stage 1** involves developing a research question appropriate for a scoping review. The following questions guided this review: 1) What is known from existing national/international research (including intervention research and programming) related to online disinformation among Black people in Africa and the African diaspora? 2) What interventions are in place to address COVID-19 disinformation in Black communities?

**Stage 2** Involves identifying relevant studies. An experienced health care librarian searched and retrieved a total of 881 articles from the following databases: Medline (1946 to present), EMBASE (1974 to present), PsycINFO (1806 to present), and Global Health (1910 to present) via OVID; Cumulative Index for Nursing and Allied Health Literature (CINAHL) (1936 to present) via EBSCOhost; Scopus (1976 to present) via Elsevier; and Cochrane Library (CENTRAL) (1993 to present) via Wiley. We derived the search strategy from three main concepts: 1) COVID-19, including variants; 2) Disinformation, misinformation, conspiracy theories, and fake news. Modes of misinformation transmission were also included, for example, social media; and 3) People of colour, specifically Black or people of African descent, or the African diaspora. For the concept of COVID-19, expert search filters were available from the University of Alberta Library (see https://guides.library.ualberta.ca/health-sciences-search-filters). No publication date, study type, or language restrictions were applied. Appendix S1 in the **Online Supplementary Document** shows full search strategies by databases. Records were managed using the systematic review software Covidence. Records from each database search were exported in complete batches and added to Covidence to remove duplicate records and facilitate screening. In total, 281 duplicate records were identified and removed, leaving 600 records for title/abstract screening. We also used Google searches to further shed light on the impact of online misinformation. Five articles from the Google search that fit the inclusion criteria were included.

**Figure 1:**
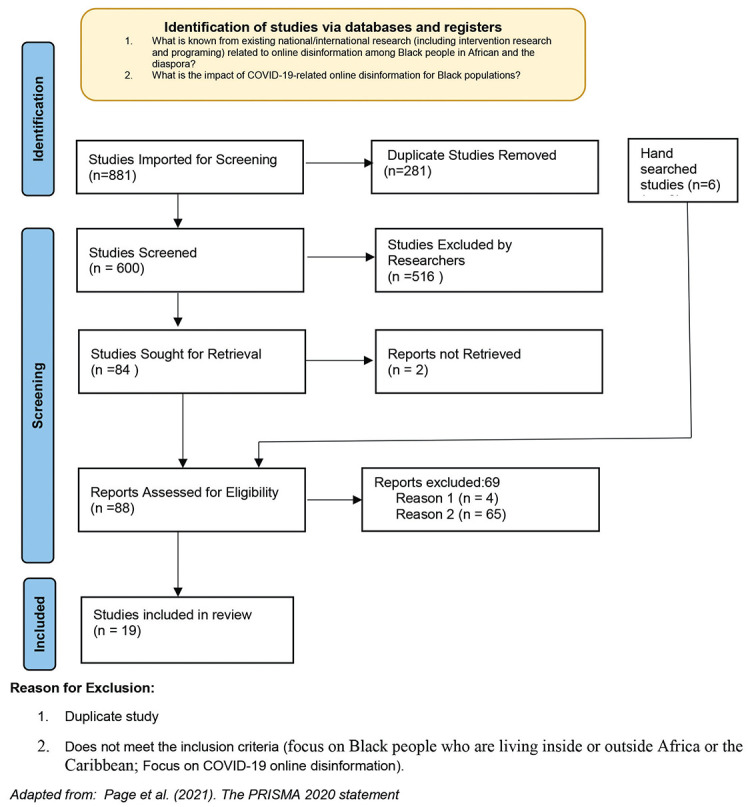
PRISMA 2020 flow diagram: online disinformation among the Black population.

**Stage 3** involves article selection. Levac et al. [25] recommend using an iterative team approach to select studies and extract data. Two reviewers independently reviewed abstracts of the 600 articles. Following this process, we excluded 516 abstracts that did not meet the inclusion criteria, which were: 1) focused on Black people living inside or outside Africa or the Caribbean; and 2) focused on COVID-19 online mis/disinformation. We were unable to access one article and hence excluded it. We also conducted a hand search, which resulted in an additional six studies being included. Two reviewers then independently reviewed the full text of the 89 included articles, excluding 70 studies not meeting the inclusion criteria and including the remaining 19 studies. A third reviewer resolved disagreements between the two reviewers at all review stages. **Figure 1** is a Preferred Reporting Items for Systematic Reviews and Meta-Analyses (PRISMA) flowchart of article identification, screening, and extraction [26].

**Stage 4** involves data charting and extraction. We documented data on an Excel spreadsheet developed by the research team. The first author (a postdoctoral fellow with a PhD in nursing) and the third author (a research assistant) extracted data from the included articles using an Excel spreadsheet approved by the research team. The extracted data included the following article characteristics: 1) title of the study; 2) purpose of the study; 3) methodology; 4) method; 5) sampling, recruitment, and selection process; 6) sample size; 7) location; 8) results; and 9) implications. The second author (a PhD prepared experienced research associate) verified the accuracy of the data extraction.

**Stage 5** involves collating, summarizing, and reporting the results. We analysed quantitative data through numerical summary by counting the number of articles and qualitative data using Braun & Clarke’s six-step thematic analysis process [27]. The first and third authors thoroughly read the included articles several times to familiarize themselves with the data. Initial codes were then generated based on the disinformation narratives identified while prioritising relevance to the research questions. Subsequently, the first author collated codes into potential themes, gathered all data relevant to each potential theme, and compared data across the coded extracts and the entire data set.

The first author also completed a numerical analysis of studies by completing a descriptive count of the number of articles based on the extracted data, such as methodology and location of the included studies. The last author (an associate professor of nursing) and the second author reviewed the codes and themes. Similarly, all authors reviewed the emerging codes and provided input. The first, second, and third authors drafted the initial report. All authors reviewed and approved the report. As a scoping review project, we did not complete a formal quality assessment of the articles. Hence, the articles included were a mix of peer-reviewed articles and grey reports that addressed COVID-19 mis/disinformation among Black populations. All authors except one are Black.

## RESULTS

All articles chosen for this review (n = 19) were written in English. Most (n = 12) were original studies with data collection responding to specific objectives. These articles indicated the purpose of the study, included detailed methods, and used sampling and recruitment strategies such as stratified purposive sampling and chain referral. Among these, six articles were quantitative, four were qualitative, two used mixed methods, and five were reports/commentaries. Most of the studies were from Africa (n = 14), with the remainder from the United States of America (n = 5). **Table 1** summarizes the characteristics of 19 articles included in this review.

**Table 1:** Main characteristics of articles included in the scoping review

Author/s	Title	Study purpose	Methodology	Method	Sampling, recruitment and selection process	Sample size	Location	Results	Implications
Adekoya & Fasae [28]	Social media and the spread of COVID-19 infodemic	To analyse the role of social media in the spread of COVID-19 infodemic in Nigeria.	Quantitative	Descriptive survey	Stratified and purposive sampling	1200	Nigeria	There was online disinformation on origin, treatment, prevention, and claims of race immunity of COVID-19 (eg, Vitamin C can cure COVID-19; The source of Covid-19 is traceable to bats; Drinking potent alcoholic drinks, exposure to high temperatures or cold weather can kill the virus; Young people or those of African origin are immune to the virus).	The COVID-19 infodemic could be managed through fact-checking information before sharing it and trusting reliable sources.
Agbor et al. [29]	Social media and management of COVID-19 in a developing county: A case of Cameroon	To discuss how to optimize public engagement to combat misinformation	commentary.	NA	NA	NA	Cameroon	Social media is very popular in Cameroon and was used to disseminate misinformation regarding the COVID-19 pandemic. However, social media could be used to increase public awareness on the availability of resources such as the government link to an online screening tool for patients who suspect they are experiencing symptoms of COVID-19.	Social media could be a useful tool to fight the pandemic in Cameroon.
Bowles et al. [30]	Countering misinformation via WhatsApp: Preliminary evidence from the COVID-19 pandemic in Zimbabwe	To understand the degree to which information from trusted social media sources impact knowledge and behaviour towards online misinformation.	Quantitative	Survey	Partner organizations distributed survey widely through email	868	Zimbabwe	Substantial numbers of participants believed in fake cures for COVID-19 spread through social media (30% believed drinking hot water and 25% believed inhaling steam cured COVID-19). There were no statistically different gender differences among the belief in fake news. However, there was evidence of knowledge and behaviour changes through the study as people began checking online information with a trusted source.	The study shows that it is possible to change knowledge and behaviour towards online misinformation when users begin checking information with a trusted source. Trust was highest in international organizations and agencies, followed by NGOs, CSOs, and government.
Collins-Dexter B. [31]	Canaries in the coal mine: COVID-19 misinformation and Black communities	To track how conspiracies and disinformation cross message boards and tech platforms and to identify predominant online narratives spreading in Black communities in the United States of America	Qualitative	Multi-site digital ethnography	None provided	None provided	USA	Misinformation identified included: Black people cannot die from COVID-19. The virus is manufactured for population control. The use of herbal remedies could contain the virus. 5 G radiation caused COVID-19 infections.	Media could avert the spread of misinformation by safeguarding accuracy, clamping down on misinformation, and supporting the dissemination of authoritative medical information in formats that speak directly to the Black community.
Dodson et al. [32]	Covid-19 vaccine misinformation and narratives surrounding Black communities on social media-First Draft	To highlight the nuance and complexity of vaccine-related narratives surrounding Black communities on social media as well as understand the dynamics of this information ecosystem.	Mixed method	Thematic analysis of the top-performing vaccine-related social media posts surrounding Black communities from November 9, 2020, to June 9, 2021.	Collected 100 most interacted-with posts from four separate entities (Unverified Facebook, Facebook groups, Instagram and Twitter accounts).	400	USA	Mistrust of COVID-19 vaccine and perception that the official institutions were coercing Black people to get vaccinated. Misinformation posted by Black antivaccine influences remains active in social media without repercussions or removal. Antivaccine content emanating from white communities has moved into Black spaces in social media, and the mistrust by some Black people of health care institutions has fuelled these narratives.	Understanding both vaccine inequity and hesitancy is essential for Black communities. Misleading vaccine posts amplify the ongoing mistrust in medical care and institutional racism. Therefore, social media companies must consistently enforce their policies against COVID-19 online misinformation. Better access to social media data by researchers and journalists could mitigate misinformation.
Emojong’ O. [33]	Fear-arousing persuasive communication and behaviour change: COVID-19 in Kenya	To understand whether fear-arousing communication (including shared over social media) contributes to behaviour change related to COVID-19.	Qualitative	12 focus groups and 9 interviews	Purposive sampling	113	Kenya	Participants were aware of COVID-19 but were generally less aware of the virus's threat and its vulnerability. Disinformation included: COVID only kills people with pre-existing medical conditions; Body temperature screening is enough to determine a person's COVID status; The disease doesn't exist; it's a government ploy to attract donor funding); The disease is not a threat to the black race; Food and alcohol give them immunity.	There is a strong relationship between perception of the COVID-19 threat and behaviour. Therefore, it is crucial to address online dis- and misinformation and disjointed communication to promote compliance with COVID-19 restrictions.
Gagliardon et al. [34]	Demystifying the COVID-19 infodemic: Conspiracies, context, and the agency of users	To assess the extent to which people bought in to the 5G and Bill Gates global conspiracies and how these conspiracies related to the political contexts in South Africa and Nigeria.	Mixed-method	Computational analysis of Twitter data sets and qualitative observation of social media conversations	Social media algorithm	713824 South African and 663 183 Nigerian Twitter users and observation of top 100 hashtags	South Africa and Nigeria	They found online misinformation about 5G infecting people with the COVID-19 virus as a means of controlling the World population. The 5G conspiracy had little buy-in, but the Bill Gates conspiracy gained traction. It was linked to resentment of the West, irritation of paternalistic attitudes and policies directed towards African countries, and resentment of corporate interests.	Leaders must take conspiracy theories seriously because they can breed mistrust. Mistrust can also vary considerably across different community contexts. Interventions into building public trust around COVID-19 restrictions and responses should consider the impact of conspiracy theories.
Goon & Okafor [35]	Curbing the COVID-19 pandemic in South Africa: Taking firmer measures and discarding fallacy theories	To show which COVID-19 measures the South Africa government has enacted and which fallacy theories are informing young people's adherence to these measures.	commentary.	N/A	N/A	N/A	South Africa	Unfounded theories circulation in the social media include: young people cannot acquire the disease nor die from it, only older, and persons with an immunocompromised and underlying illness are vulnerable to COVID-19 and could die from the disease. Misrepresentation of facts that particular races are immune to COVID-19 and COVID-19 not thriving in hot temperatures.	There is a need for collaboration and a disciplined approach from the government, the private sector, and the scientific community.
Hassan I [36]	The dual threat of a virus and a fake news Epidemic		Opinion	NA	NA	NA	Nigeria	Cures such as constant sex, sitting in the sun, or African blood is immune to the virus. The use of chloroquine will cure the virus	The government could contain COVID-19 misinformation by communicating transparent information to its citizens.
Igbinovia et al. [37]	Information literacy competence in curtailing fake news about the COVID-19 pandemic among undergraduates in Nigeria	To analyse the effect of information literacy competency (LIC) in slowing the spread of fake news among library and information science (LIS) undergraduate students in Nigeria.	Quantitative	Survey with descriptive analysis	Stratified random sampling	138	Nigeria	LIS undergraduates have high levels of information literacy competency (ILC) and are well-positioned to stop/slow the spread of fake news. However, being over inundated with COVID-19 information decreased participants' abilities to discern fake news from correct data. Disinformation included: If you can hold your breath for 10s without discomfort, you don't have COVID-19; COVID-19 is a big man's disease and does not affect the poor; Frequent alcohol intake will destroy COVID-19 in the body system; A saline solution of warm water and salt or vinegar will remedy COVID-19; There is no COVID-19 in Nigeria; 5G is the cause of COVID-19; Constant exposure to sunlight will prevent the transmission of COVID-19.	Children should receive training on ILC skills in schools because fighting the infodemic is a critical skill.
Lipfert K [38]	Fake news and coronavirus, two global pandemics to counter		Report	NA	NA	NA	Cote d'Ivoire	Barack Obama asks Africans not to accept vaccines that will come from Europe	People should first pay close attention to the details, make sure it is a reliable source, check the official websites of the social media accounts and diversify news sources by consulting other articles on the same subject
Ndoro, T. [39]	Coronavirus Misinformation Going Viral on Social Media in Africa	To demystify misinformation in African countries	Report	Na	NA	NA		African people are used as guinea pigs to test a new coronavirus vaccine. In Kenya-Black, people cannot contract coronavirus, drinking tea. Nigeria-shaving beards prevent coronavirus, drinking pepper soup.	Fears about vaccines are not uncommon among many communities, indicating the need to educate the public about COVID-19 virus and vaccine.
NPR [40]	An Anti-Vaccine Film Targeted to Black Americans Spreads False Information	To report online misinformation targeted at Black people	Report	NA	NA	NA	USA	Children Health Defence, an anti-vax group, released a movie that discredits vaccines. The film targets Black people. It includes people of colour talking about COVID-19 as “propaganda” and not trusting the vaccine. Despite the efforts to improve the uptake of the COVID-19 vaccine, media coverage has capitalized on talks about the historical experimentation in America, Africa, and other parts of the world.	The ideal strategy to curb the spread of online misinformation is to cut it off from the source to prevent its spread. We need to try and make people aware of the false and misleading claims.
Obi-Ani et al. [41]	Social media and the Covid-19 pandemic: Observations from Nigeria	The purpose of this study was to interrogate the role social media plays in curtailing and aiding the spread of news regarding the Pandemic	Quantitative	Questionnaires were utilized for the primary data and document analysis for the secondary data	The questionnaires were shared on different online platforms and through personal contact.	100	Nigeria	They found online misinformation about the prevention or treatment of COVID-19, such as using hot water, garlic, or chloroquine. Religious leaders spread rumours about the COVID-19 virus being used as a ploy to install the 5G technology, and the Chinese government's test kits are infected with COVID-19. Celebrities with many followers fuelled the spread of misinformation. To curb the spread of online misinformation is to cut it off from the source to prevent spread. We need to try and make people aware of the false and misleading claims.	Social media is a good tool, but it can be divisive and distrustful; therefore, people should endeavour to conduct personal research on sources before believing it. Consequently, it is necessary to enlighten people to verify social media information to debunk fake news.
Omaka-Amari et al. [42]	Coronavirus (COVID-19) Pandemic in Nigeria: Preventive and Control Challenges within the First Two Months of Outbreak.	To determine the factors that posed as challenges to the prevention and control of COVID 19	Quantitative	Analysis of data from the daily updates of Nigeria Centre for Disease Control (NCDC) as well as other resources about COVID 19.	N/A	N/A	Nigeria	Social media spread misinformation about masks claiming people will react to them. Misinformation that the COVID-19 vaccine will wipe out Nigerians	The myths and misconceptions regarding COVIID 19 can be alleviated through evidence-based campaigns using all sources of information.
Ross J. [43]	Corona virus misinformation crosses divides to infect black social media	Presents highlights on coronavirus disinformation propagated by major black influences ion social media	Report	NA	NA	NA	USA	Distrust allows misinformation to flourish in social media. Increased rumours among Black people about the use of hydroxychloroquine to treat COVID-19. They capitalized on historical experiments on black people such as the Tuskegee experiment, birth control experiment, and involuntary sterilization programs in Puerto Rico and Native American women, and the poor. Belief that COVID-19 virus was manufactured in China and USA laboratories, vaccine-related money-scheme, COVID-19 virus is spread by cell phone technology, and Black people are immune to COVID-19.	Black social media spreading disinformation to their black audience contributes to the vaccine hesitancy among African Americans.
Schmidt et al. [44]	Myths, misconceptions, othering and stigmatizing responses to Covid-19 in South Africa: A rapid qualitative assessment	The aim of this study is to investigate South African communities' construction of myths and misconceptions about who is most vulnerable to Covid-19 and how at times these beliefs inform a discourse of stigmatization and othering.	Qualitative	Interviews and one focus group discussions	Purposive sampling	60	South Africa	The study found that information circulated on social media such as drinking hot water and eating corn and porridge to cure COVID-19, the virus was manufactured and is transmitted via 5G technology, and COVID-19 testing kits are contaminated. These false beliefs instigated confusion, fear, and panic, which influenced public responses to screening and testing campaigns and contributed to the construction of misconceptions, othering and stigmatizing responses to Covid-19	The study findings highlight the importance of developing communication materials adapted to specific communities to help reduce misconceptions, othering, and stigmatization around Covid-19. Myths, misconceptions, othering, and stigmatizing responses to Covid-19 in South Africa can hamper efforts to mitigate the transmission of the disease. It might impact public health efforts to test, track, and trace those infected with Covid-19.
Stacey K. [45]	Anti-vaxxers are targeting black people, warns US Covid adviser	Reports online misinformation targeted at Black people	Report	NA	NA	NA	USA	Anti-vaxxers target black people using historical scandals such as the Tuskegee study in 1932 and Smallpox infested blankets. Black people have lower vaccination rates, although they are predominantly affected by COVID-19.	We need to clamp down on COVID-19 misinformation to increase vaccine acceptance.
Talabi et al. [46]	Effect of a social media based counselling intervention in countering fake news on COVID-19 vaccine in Nigeria	The objective of this study was to test the effectiveness of a social media-based counselling intervention in countering the impact of fake news on COVID-19 vaccine among social media users in Nigeria.	Quantitative	Quasi experiments with use of Questionnaires. -	Respondent-driven sampling (RDS) chain referrals.	470 respondents took part in the first experiment.	Nigeria	Fake news through social media negatively impacts public perception regarding the COVID-19 vaccine. Disinformation includes: COVID-19 vaccine will alter people DNA, people who receive the vaccine will die in two years, COVID-19 vaccine was developed in a hurry, COVID-19 vaccine cannot be trusted, the vaccine has serious negative side effects, COVID-19 vaccine contains a chip, COVID-19 vaccine is not effective, and COVID-19 vaccine is not meant for Africa countries.	Counselling through social media could effectively counter fake news within the context of health promotion.
Tibbels et al. [47]	Real-Time Tracking of COVID-19 Rumors Using Community-Based Methods in Côte d'Ivoire	To summarize the multipronged community-based approach to rumour collection, the process of coding and managing rumour submissions, and summarize user feedback and lessons learned for tracking rumours during a public health emergency.	Qualitative (real-time monitoring)	Rumours were submitted through a hotline or WhatsApp line. Both community contributors and teleoperators worked to receive rumours.	Purposive sampling	1,757	Cote d'Ivoire	Rumours and misinformation about the lack of trust in the government and the international public health response are lacking. There was the denial of the reality of the virus or case estimates. They believed the virus was intentionally manufactured in a laboratory to kill people and that the danger was now over. Belief particular food and herbal remedies will cure COVID-19, and relational perception of the severity of the disease (kills faster than Ebola, malaria is worse than COVID, etc.).	The use of real-time rumour tools could enable a public health response and interventions that promote effective communications.

Several studies reported that most Black people turned to social media for information following the global lockdown during the COVID-19 pandemic, including the use of platforms such as WhatsApp, Facebook, Twitter, YouTube, and Instagram, among others. Exposure to disinformation occurred in different ways, including the intentional spread of false information, inappropriate treatment advice, misrepresented pictures and videos, and reconfigured misinformation [28]. Additionally, we found information related to ways that dis/misinformation is spread, including vehicles used for dissemination. We present the results under six themes: 1) Theories on the origin of COVID-19 (n = 6); 2) Theories on transmission, prevention, and treatment of COVID-19 (n = 11); 3) Theories on speculation of race immunity against COVID-19 (n = 3); 4) Distrust in government and health organizations (n = 7); 5) Intervention research and programming (n = 3); 6) Knowledge about how dis/misinformation is spread through online vehicles (n = 10)

### Theories on the origin of COVID-19

Articles discussing theories on the origin of COVID-19 were consistent in reporting that Black communities believed rumours that the COVID-19 virus was created as a biological weapon [31,34,37,43,44,47]. The frequently cited origin of the COVID-19 virus was that it was: man-made for population control [31,34]; manufactured in China and US laboratories for vaccine-related monetary schemes [34,43,44]; linked to some of the world elite, resentment of the West, and authoritarian attitudes and policies directed towards African countries [34]; or caused by fifth-generation technology for broadband cellular networks (5G) radiation [31,37,44]. A study that tracked real-time rumours on COVID-19 found people in Cote d’Ivoire believed the virus was intentionally manufactured in a laboratory to kill people, with the danger now being over [47].

### Theories on transmission, prevention, and treatment of COVID-19

A considerable portion of the Black population believed in disinformation about the transmission, prevention, and treatment of COVID-19 spread through social media [30,31,33,41,42,44,47]. For instance, many purported the belief that following alternative prevention and treatment methods would prevent or cure COVID-19; these included: drinking hot water [30,41,44], inhaling steam [30], using herbal remedies [31,39,41,47], eating certain foods [33,37,44,47], drinking alcohol [28,37,44,47], drinking a saline solution of warm water and salt or vinegar [37], using drugs such as hydroxychloroquine [36,41,43], constant exposure to sunlight [37] and hot temperatures [28,35] or cold temperatures [28]. Some believed that engaging in constant sex and sitting in the sun [36] or shaving their beard [39] would prevent the transmission of COVID-19.

Further, disinformation about perceived transmission included the notions that young people cannot acquire the disease, with only older persons and people who are immunocompromised and have underlying illnesses being vulnerable [35], and that COVID-19 is a “big man's disease” and does not affect the poor [37]. Some disinformation undermined the preventative measures put in place to combat COVID-19, including ideas that: the test kits are infected with COVID-19 by the Chinese Government [41,44]; people will have adverse reactions from the use of masks, including difficulty in breathing [42]; and the COVID-19 vaccine will wipe out Nigerians [42].

### Theories on speculation of race immunity against COVID-19

Claims of race-based resilience against COVID-19 among Black people flooded social media at the beginning of the pandemic [30,31,35,43]. In the USA, Collins-Dexter and Ross [31,43] proposed that Black people are immune and cannot die from COVID-19 circulated on social media even when the American Government reported that Black people were disproportionately affected by the pandemic.

Similarly, a qualitative study in Kenya to understand whether fear-arousing communications contribute to behaviour change related to COVID-19 found the participants perceived the disease as not being a threat to Black people [33] and that Black people could not contract the virus [28,36,39]. In South Africa, Goon and Okafor [35] noted misrepresentation of facts that some races (including Blacks) were immune to COVID-19. Furthermore, in the USA, Ross [43] reported that Black worshippers believed they could not be infected at church while attending in-person services.

### Distrust in governments and health organizations

In Cameroon, the public's lack of trust in government authorities was the primary driver of misinformation [29]. Rumours and misinformation about the lack of confidence in the government, as well as the international public health response, led people to deny the reality of case estimates of COVID-19 and even relate the severity of the disease to other infections such as Ebola and malaria [47]. Some believed COVID-19 did not exist in their country and that it was a government ploy to obtain money from donors [33,37]. Some religious leaders spread rumours about COVID-19 being used as a tactic to install 5G technology [41]. While anti-vaccine groups capitalized on the historical experiments on Black people, such as the Tuskegee experiment, birth control experiment, and involuntary sterilization programs in Puerto Rico and on Native American women as well as the poor to deter Black people from receiving the COVID-19 vaccine [43,45].

Other rumours with serious negative implications for people’s perception of COVID-19 included notions that persons who receive the vaccine will die within two years, that the COVID-19 vaccine will alter people’s DNA, that the vaccine was developed in a hurry and cannot be trusted, that the vaccine has serious negative side effects, that the vaccine contains a chip, that the vaccine is ineffective, and that the vaccine is unsuitable for Black people in general [46]. Some indicated the former president of the USA urged Black African people not to accept vaccines from Europe [38].

### Intervention research and programming

This review identified three studies that sought to identify measures that could address COVID-19 misinformation [30,46,47]. To counter misinformation via WhatsApp in Zimbabwe [30], researchers partnered with established and trusted organizations such as Internews and Kubatana. Each week, the organizations disseminated a short WhatsApp message about COVID-19 (mode of transmission and prevention measures). The organizations could reach participants, deter misconceptions through WhatsApp, and correct participant behaviours [30]. Another study used quasi-experiments to test the effectiveness of a social media-based counselling intervention in countering the impact of fake news on the COVID-19 vaccine among social media users in Nigeria [46]. These researchers exposed a group of participants to fake news about COVID-19 through a WhatsApp group. Half of these participants were then exposed to social media-based counselling interventions about the importance of the COVID-19 vaccine. The study found participants who had been exposed to counselling scored higher regarding behaviour intention to make themselves available for COVID-19 vaccination [46].

Similarly, Côte d'Ivoire utilized real-time rumour-tracking of COVID-19 using community-based methods to determine people's perceptions of COVID-19 [47]. Community contributors submitted rumours to a designated WhatsApp communication or the Ministry of Digital Health number. Researchers shared the data from the rumour-tracking system with the National Risk Communication Technical Working Group (RCTWG), who then developed strategic communications for the public to counter the rumours. The study concluded that real-time rumour tools could enable a public health response and interventions that promote effective communication [47].

### Knowledge about how dis/misinformation is spread through online vehicles

In addition to the types of dis/misinformation being spread, a sixth theme emerged through this review: ways that dis/misinformation is spread through online vehicles. While one study [41] emphasized ways in which individuals are purposefully abusing social media platforms to instigate panic, other studies show false information is sometimes spread in error without malicious intent, more often consisting of misinformation rather than disinformation [28]. This often happens when people share posts created by other people [29]. This may explain the emergence of an infodemic related to COVID-19, as much of the spread of dis/misinformation occurs through information-seeking by users on how to navigate the crisis [14]. We also recognize that fake news related to COVID-19 often utilizes conspiracy theories and rumours that play on existing relationships of distrust, often incorporating distrust in government [30,43,47]. This has been especially relevant with respect to vaccine hesitancy [48].

Most participants in one study [28] agreed that digital literacy, government regulation of social media, and educational videos about COVID-19 were important factors in managing the COVID-19 infodemic. Preventative digital literacy has also been cited as an important weapon against COVID-19 online dis/misinformation [37]. In another case [46], reactive counselling was shown to mitigate against the effects of fake news.

## DISCUSSION

This scoping review synthesized knowledge about online disinformation and misinformation related to COVID-19 among Black people around the world. Such a review is both important and pressing as it offers insight into the public health implications of false information during the ongoing global pandemic. The findings indicate Black people are accessing and often sharing online disinformation and misinformation primarily through social media platforms such as WhatsApp, Facebook, Twitter, YouTube, and Instagram. Dis/misinformation concerns were related to the origin of COVID-19, transmission, prevention, treatment, assertions of race immunity to the virus, distrust in government and health organizations, and intervention research and programming.

As COVID-19 was only declared a pandemic in early 2020, our findings do not corroborate or dispute previous reviews because none exist. Instead, they shed light on a newly emerging field of study. The first theme we identified concerned dis/misinformation regarding the origins of COVID-19. Our results show dis/misinformation asserts the virus was purposefully created, either as a biological weapon meant to control population growth or as a part of a scheme to enrich companies through the sale of vaccines. These rumours were particularly salient in studies from African countries, where resentment of Western elites already existed from historical exploitation.

The second was a theme related to transmission, prevention, and treatment of COVID-19 and is perhaps the most relevant for public health policies. Incorrect theories suggested people could prevent or treat symptoms by drinking hot water, inhaling steam, eating certain foods, drinking alcohol, and ingesting drugs such as hydroxychloroquine. Most of these prevention and treatment strategies utilised temperature, advising that either extreme cold or heat kills the virus. These findings are particularly troubling to public health measures because they may prevent people from accessing proper medical services when sick, possibly exposing others to risk, and in some cases, may themselves have detrimental health impacts.

Assumptions that the virus only targets the old, immunocompromised, and rich may leave many working-class and poorer youth extremely vulnerable to illness, especially if people are accessing these ideas in conjunction with dis/misinformation related to prevention and treatment and distrust of effective measures such as test kits, masks, and vaccines. Non-racially disaggregated studies concerning COVID-19-related conspiracy theories in the United States show this dis/misinformation can prompt people to reject expert advice, including accepting vaccines, and to engage in risky behaviour by flouting public health measures [48-50]. However, some evidence on the popularity of conspiracy theories has been overstated [49]. Further research could investigate the degree to which dis/misinformation related to COVID-19 translates into action.

Next, we found that claims of race-based resilience against the virus compounded dis/misinformation around transmission, prevention, and treatment. Claims that Black people are immune to COVID-19 directly contradict evidence that Black people in Canada and the United States were/are disproportionately impacted by the pandemic [51-53]. Assumptions of race-based immunity could prevent some Black people from accessing vaccines against COVID-19, which would further deepen racial health inequities in the United States [54]. While race-based data concerning vulnerabilities to COVID-19 are less available in Canada, for instance, evidence suggests Black communities have been more vulnerable to health disparities in general and related to the virus specifically [55].

Fourth, this review showed that dis/misinformation often targets Black people’s distrust in government and national and international public health organizations. This theme is directly related to theme two, in that distrust in authority is linked to understandings of prevention and treatment of the virus. Those who distrust government and medical experts may be less open to follow the recommended effective prevention and treatment strategies and more likely to attempt to self-medicate symptoms and be resistant to test kits, masks, and vaccines. This issue is directly connected to information meant to make people think the virus was purposefully created and, in African countries, may be particularly salient due to pre-existing relationships with Western exploitation. Similarly, content reverberates when it comes from religious leaders, hence Hong and Handal [56] have instilled the importance of science, religions, and governments working together for the common well-being of all.

A theme of intervention research and programming also emerged. Several studies discussed ways that governments and organizations may respond to online dis/misinformation about COVID-19. This is an area of extreme importance and there is a demand for further research into strategies to combat the “dual threat” of fake news alongside the virus [36]. Responses to fake news gained considerable traction globally in the face of rising populism [57-59]. Research into this specific form of fake news and its impact on specific populations may be well advised to draw on lessons already learned in this field. Finally, we presented findings related to the ways that dis/misinformation is being spread through online vehicles, indicating these vehicles are primarily social media platforms.

Current knowledge indicates some disagreement about the infodemic spread. As noted, some assert this problem is being created purposefully, while others report information-seeking behaviour and fear driving people to share fake news without malicious intent. Both realities likely co-exist. The democratization of online news and information through social media has created the conditions within which fake news is able to spread quickly and widely [60]. However, discrepancies in how we understand the root causes of the creation and spread of dis/misinformation will have crucial implications for how governments, service providers, and organizations respond to this issue.

Our results show a lack of qualitative analysis of online dis/misinformation related to COVID-19 impacting Black people, as only four qualitative studies have been conducted globally [33,41,44,47]. Knowledge has so far been overwhelmingly produced in African countries. Only five of the included studies [31,32,40,43,45] are from outside of Africa and all are based in the United States. There is a gap concerning knowledge in other settings globally, including Canada. There are opportunities in North American and other Western countries with large Black populations to draw lessons learned in African countries to address domestic racially targeted COVID-19 disinformation and misinformation.

Additionally, knowledge is lacking with respect to the impact and implications of online dis/misinformation, including the extent to which false information informs people’s adherence to public health measures. Though data are emerging concerning the psychological and practical impacts of online dis/misinformation related to COVID-19 [61-63], a gap remains concerning racially disaggregated data. Future research may consider the theoretical and practical implications of disinformation and misinformation related to COVID-19 on Black people and other demographics globally.

## CONCLUSIONS

This review sought to synthesize current research and identify instances of COVID-19 online disinformation among Black populations in Africa and the African diaspora, identify measures to address COVID-19 disinformation in Black communities, and identify gaps for future research on pandemic disinformation among Black people. Existing knowledge surrounding online dis/misinformation related to COVID-19 can be organized into six themes: theories on the origin of COVID-19; theories on transmission, prevention, and treatment of COVID-19; theories on speculation of race immunity against COVID-19; distrust in government and health organizations; intervention research and programming; and knowledge about how dis/misinformation is spread through online vehicles. The findings suggest a need for more qualitative data concerning Black people’s experiences with online dis/misinformation related to COVID-19.

## Additional material


Online Supplementary Document
