# Supplementary material for: A scoping review of COVID-19 online mis/disinformation in Black communities
Source: J Glob Health. 2022 Jul 23;12:05026. doi: 10.7189/jogh.12.05026 (PMC9304926; doi:10.7189/jogh.12.05026)
Supplement: Online Supplementary Document [file jogh-12-05026-s001.pdf]

Appendix 1 - Search strategies by database

Ovid MEDLINE(R) ALL <1946 to September 27, 2021>
Date of search: September 28, 2021

1 (((exp Coronavirus/ or exp Coronavirus Infections/ or (coronavirus* or corona virus* or OC43 or NL63 or 229E or HKU1 or HCoV* or ncov* or covid* or sars-cov* or sarscov* or Sars-coronavirus* or Severe Acute Respiratory Syndrome Coronavirus*).mp.) and 20190601:20301231.(ep).) not (SARS or SARS-CoV or MERS or MERS-CoV or Middle East respiratory syndrome or camel* or dromedar* or equine or coronary or coronal or covidence* or covidien or influenza virus or HIV or bovine or calves or TGEV or feline or porcine or BCoV or PED or PEDV or PDCoV or FIPV or FCoV or SADS-CoV or canine or CCov or zoonotic or avian influenza or H1N1 or H5N1 or H5N6 or IBV or murine corona*).mp.) or Covid-19/ or (covid or covid19 or 2019-ncov or ncov19 or ncov-19 or 2019-novel CoV or sars-cov2 or sars-cov-2 or sarscov2 or sarscov-2 or Sars-coronavirus2 or Sars-coronavirus-2 or SARS-like coronavirus* or coronavirus-19 or ((novel or new or nouveau) adj2 (CoV or nCoV or covid or coronavirus* or corona virus or Pandemi*2)) or (variant* adj2 (India* or "South Africa*" or UK or English or Brazil* or alpha or beta or delta or gamma or kappa or lambda or "P.1" or "C.37")) or ("B.1.1.7" or "B.1.351" or "B.1.617.1" or "B.1.617.2")).mp. 188832

2 exp African Continental Ancestry Group/ 91750
3 (black* or african* or caribbean or afro* or "person of colo?r" or "people of colo?r" or colo?red or "dark-skin*" or racial* or race? or ((racial or ethnic) adj2 minorit*)).mp. 492578
11 exp africa/ or exp africa, northern/ or exp "africa south of the sahara"/ or exp africa, central/ or exp africa, eastern/ or exp africa, southern/ or exp africa, western/ 289967
12 exp caribbean region/ or exp west indies/ 31780
13 (algeria* or angola* or benin* or Botswana* or "burkina faso" or Burundi or Cameroon or "Cape Verde" or "Central African Republic" or chad or Comoros or Congo* or "cote d'ivoire" or "Ivory Coast" or Djibouti or Egypt* or Guinea* or Eritrea or Ethiopia* or Gabon or Gambia* or ghana* or Kenya* or Lesotho or Liberia* or Libya* or Madagascar or Malawi* or mali* or Mauritania* or Mauritius or Morocco or Mozambique or Namibia* or niger or nigeria* or rwanda* or "sao tome and principe" or senegal* or seychelles or "sierra leone" or somalia* or "south africa" or sudan or swaziland or tanzania* or togo or Tunisia* or uganda* or Zambia* or Zimbabwe*).ti,ab. 1039731
14 ("Dominican Republic" or Haiti* or Jamaica* or Barbados or Dominica or Grenada or "Saint Lucia" or trinidad* or Bahamas).mp. 17975
15 Oceanic Ancestry Group/ 11141
16 ((indigenous or aboriginal* or aborigines or "First Nations" or "first peoples") adj3 australia*).mp. 5025
17 or/9-16 1580454
18 1 and 4 and 8 and 17 144

OVID Embase <1974 to 2021 September 27>
Date of search: September 28, 2021

1 ((exp Coronavirus/ or exp Coronavirus Infections/ or (coronavirus* or corona virus* or OC43 or NL63 or 229E or HKU1 or HCoV* or ncov* or covid* or sars-cov* or sarscov* or Sars-coronavirus* or Severe Acute Respiratory Syndrome Coronavirus* or D614G).mp.) not (SARS or SARS-CoV or MERS or MERS-CoV or Middle East respiratory syndrome or camel* or dromedar* or equine or coronary or coronal or covidence* or covidien or influenza virus or HIV or bovine or calves or TGEV or feline or porcine or BCoV or PED or PEDV or PDCoV or FIPV or FCoV or SADS-CoV or canine or CCov or zoonotic or avian influenza or H1N1 or H5N1 or H5N6 or IBV or murine corona*).mp.) or coronavirus disease 2019/ or (((pneumonia or covid* or coronavirus* or corona virus* or ncov* or 2019-ncov or sars*).mp. or exp pneumonia/) and Wuhan.mp.) or ("coronavirus disease 2019" or 2019-ncov or ncov19 or ncov-19 or 2019-novel CoV or severe acute respiratory syndrome coronavirus 2 or sars-cov2 or sars-cov-2 or sarscov2 or sarscov-2 or Sars-coronavirus2 or Sars-coronavirus-2 or SARS-like coronavirus* or coronavirus-19 or covid19 or covid-19 or "covid 2019" or "B.1.1.7" or "B.1.351" or "B.1.617.1" or "B.1.617.2" or (variant* adj2 (India* or "South Africa*" or UK or English or Brazil* or alpha or beta or delta or gamma or kappa or lambda or "P.1" or "C.37")) or ((novel or new or nouveau) adj2 (CoV or nCoV or coronavirus* or corona virus))).mp. 204178
2 exp misinformation/ 1896
3 (misinform* or mis-inform* or disinformation or misperception* or misconception* or misinterpret* or "fake news" or infodemic* or info-demic or conspirac* or ((information or fact? or science or scienti* or claim* or knowledge or source* or evidence or statement* or guide*) adj4 (false* or mislead* or misled or alternative* or deceptive or deception or poor or bad or wrong or correct* or incorrect or trustworth* or untrustworth* or distrust* or mistrust* or confidence or confident or accurac* or accurate or inaccurate or valid* or invalid* or reliabl* or unreliable)) or rumor* or hoax* or myth* or "urban legend*" or gossip or fallac* or "anti-vaxx*" or "anti-scien*" or "false dichotom*" or pseduoscience or pseudo-science).mp. 234388
4 2 or 3 234388
5 social media/ 29904
6 exp blogging/ 485
7 internet access/ 1098
8 internet use/ 266
9 social network/ or online social network/ 19753
10 ("social media" or "social site*" or Facebook or twitter or tweet* or instagram* or "tik Tok" or YouTube* or Tumblr or reddit or Qzone or snapchat or "snap chat" or Pinterest or influencer* or "broadcast media" or news* or blog* or internet or digital* or online or on-line or web* or bot or bots or cyber* or "social network*" or "commercial media" or email or clickbait* or "click-bait*" or "mass media").mp. 911055
11 or/2-10 1121616
12 exp african/ or exp central african/ or exp east african/ or exp north african/ or exp southern african/ or exp west african/ 30604
13 exp black person/ 120525
14 exp caribbean/ 4162

15 exp Caribbean Islands/ 32344
16 (black* or african* or caribbean or afro* or "person of colo?r" or "people of colo?r" or colo?red or "dark-skin*" or racial* or race? or ((racial or ethnic) adj2 minorit*)).mp. 772952
17 (algeria* or angola* or benin* or Botswana* or "burkina faso" or Burundi or Cameroon or "Cape Verde" or "Central African Republic" or chad or Comoros or Congo* or "cote d'ivoire" or "Ivory Coast" or Djibouti or Egypt* or Guinea* or Eritrea or Ethiopia* or Gabon or Gambia* or ghana* or Kenya* or Lesotho or Liberia* or Libya* or Madagascar or Malawi* or mali* or Mauritania* or Mauritius or Morocco or Mozambique or Namibia* or niger or nigeria* or rwanda* or "sao tome and principe" or senegal* or seychelles or "sierra leone" or somalia* or "south africa" or sudan or swaziland or tanzania* or togo or Tunisia* or uganda* or Zambia* or Zimbabwe*).ti,ab. 1342399
18 ("Dominican Republic" or Haiti* or Jamaica* or Barbados or Dominica or Grenada or "Saint Lucia" or trinidad* or Bahamas).mp. 20495
19 indigenous australian/ 1107
20 ((indigenous or aboriginal* or aborigines or "First Nations" or "first peoples") adj3 australia*).mp. 6185
21 or/12-20 2075733
22 1 and 4 and 11 and 21 306

OVID APA PsycInfo <1806 to September Week 3 2021>
Date of search: September 28, 2021

1 ((exp Coronavirus/ or (coronavirus* or corona virus* or OC43 or NL63 or 229E or HKU1 or HCoV* or ncov* or covid* or sars-cov* or sarscov* or Sars-coronavirus* or Severe Acute Respiratory Syndrome Coronavirus* or D614G).mp.) not (SARS or SARS-CoV or MERS or MERS-CoV or Middle East respiratory syndrome or camel* or dromedar* or equine or coronary or coronal or covidence* or covidien or influenza virus or HIV or bovine or calves or TGEV or feline or porcine or BCoV or PED or PEDV or PDCoV or FIPV or FCoV or SADS-CoV or canine or CCov or zoonotic or avian influenza or H1N1 or H5N1 or H5N6 or IBV or murine corona*).mp.) or Covid-19/ or (((pneumonia or covid* or coronavirus* or corona virus* or ncov* or 2019-ncov or sars*).mp. or exp pneumonia/) and Wuhan.mp.) or ("coronavirus disease 2019" or 2019-ncov or ncov19 or ncov-19 or 2019-novel CoV or severe acute respiratory syndrome coronavirus 2 or sars-cov2 or sars-cov-2 or sarscov2 or sarscov-2 or Sars-coronavirus2 or Sars-coronavirus-2 or SARS-like coronavirus* or coronavirus-19 or covid19 or covid-19 or covid 2019 or "B.1.1.7" or "B.1.351" or "B.1.617.1" or "B.1.617.2" or (variant* adj2 ("South Africa*" or UK or English or Brazil* or alpha or beta or delta or gamma or kappa or lambda or "P.1" or "C.37")) or ("B.1.1.7" or "B.1.351" or "B.1.617.1" or "B.1.617.2") or ((novel or new or nouveau) adj2 (CoV or nCoV or coronavirus* or corona virus))).mp. 9730
2 (misinform* or mis-inform* or disinformation or misperception* or misconception* or misinterpret* or "fake news" or infodemic* or info-demic or conspirac* or ((information or fact? or science or scienti* or claim* or knowledge or source* or evidence or statement* or guide*) adj4 (false* or mislead* or misled or alternative* or deceptive or deception or poor or bad or wrong or

correct* or incorrect or trustworth* or untrustworth* or distrust* or mistrust* or confidence or confident or accurac* or accurate or inaccurate or valid* or invalid* or reliabl* or unreliable)) or rumor* or hoax* or myth* or "urban legend*" or gossip or fallac* or "anti-vaxx*" or "anti-scien*" or "false dichotom*" or pseduoscience or pseudo-science).mp. 97965

3 exp social media/ or blog/ or online community/ or exp websites/ 23515

4 internet usage/ or computer searching/ 4406

5 ("social media" or "social site*" or Facebook or twitter or tweet* or instagram* or "tik Tok" or YouTube* or Tumblr or reddit or Qzone or snapchat or "snap chat" or Pinterest or influencer* or "broadcast media" or news* or blog* or internet or digital* or online or on-line or web* or bot or bots or cyber* or "social network*" or "commercial media" or email or clickbait* or "click-bait*" or "mass media").mp. 275907

6 3 or 4 or 5 276351

7 african cultural groups/ or blacks/ 57363

8 (black* or african* or caribbean or afro* or "person of colo?r" or "people of colo?r" or colo?red or "dark-skin*" or racial* or race? or ((racial or ethnic) adj2 minorit*)).mp. 239576

9 (algeria* or angola* or benin* or Botswana* or "burkina faso" or Burundi or Cameroon or "Cape Verde" or "Central African Republic" or chad or Comoros or Congo* or "cote d'ivoire" or "Ivory Coast" or Djibouti or Egypt* or Guinea* or Eritrea or Ethiopia* or Gabon or Gambia* or ghana* or Kenya* or Lesotho or Liberia* or Libya* or Madagascar or Malawi* or mali* or Mauritania* or Mauritius or Morocco or Mozambique or Namibia* or niger or nigeria* or rwanda* or "sao tome and principe" or senegal* or seychelles or "sierra leone" or somalia* or "south africa" or sudan or swaziland or tanzania* or togo or Tunisia* or uganda* or Zambia* or Zimbabwe*).ti,ab. 63798

10 ("Dominican Republic" or Haiti* or Jamaica* or Barbados or Dominica or Grenada or "Saint Lucia" or trinidad* or Bahamas).mp. 4932

11 ((indigenous or aboriginal* or aborigines or "First Nations" or "first peoples") adj3 australia*).mp. 1952

12 7 or 8 or 9 or 10 or 11 295856

13 1 and 2 and 6 and 12 11

OVID Global Health <1910 to 2021 Week 38>
Date of search: September 28, 2021

1 ((coronavirus* or corona virus* or OC43 or NL63 or 229E or HKU1 or HCoV* or ncov* or covid* or sars-cov* or sarscov* or Sars-coronavirus* or Severe Acute Respiratory Syndrome Coronavirus* or D614G) not (SARS or SARS-CoV or MERS or MERS-CoV or Middle East respiratory syndrome or camel* or dromedar* or equine or coronary or coronal or covidence* or covidien or influenza virus or HIV or bovine or calves or TGEV or feline or porcine or BCoV or PED or PEDV or PDCoV or FIPV or FCoV or SADS-CoV or canine or CCov or zoonotic or avian influenza or H1N1 or H5N1 or H5N6 or IBV or murine corona*)).mp. 25819

2 (pneumonia or covid* or coronavirus* or corona virus* or ncov* or 2019-ncov or sars* or (pneumonia and wuhan) or ("coronavirus disease 2019" or 2019-ncov or ncov19 or ncov-19 or

2019-novel CoV or severe acute respiratory syndrome coronavirus 2 or sars-cov2 or sars-cov-2 or sarscov2 or sarscov-2 or Sars-coronavirus2 or Sars-coronavirus-2 or SARS-like coronavirus* or coronavirus-19 or covid19 or covid-19 or "covid 2019" or "B.1.1.7" or "B.1.351" or "B.1.617.1" or "B.1.617.2" or (variant* adj2 (India* or "South Africa*" or UK or English or Brazil* or alpha or beta or delta or gamma or kappa or lambda or "P.1" or "C.37")) or ((novel or new or nouveau) adj2 (CoV or nCoV or coronavirus* or corona virus)))).mp. 104316

3 1 or 2 104327

4 (misinform* or mis-inform* or disinformation or misperception* or misconception* or misinterpret* or "fake news" or infodemic* or info-demic or conspirac* or ((information or fact? or science or scienti* or claim* or knowledge or source* or evidence or statement* or guide*) adj4 (false* or mislead* or misled or alternative* or deceptive or deception or poor or bad or wrong or correct* or incorrect or trustworth* or untrustworth* or distrust* or mistrust* or confidence or confident or accurac* or accurate or inaccurate or valid* or invalid* or reliabl* or unreliable)) or rumor* or hoax* or myth* or "urban legend*" or gossip or fallac* or "anti-vaxx*" or "anti-scien*" or "false dichotom*" or pseduoscience or pseudo-science).mp. 35690

5 ("social media" or "social site*" or Facebook or twitter or tweet* or instagram* or "tik Tok" or YouTube* or Tumblr or reddit or Qzone or snapchat or "snap chat" or Pinterest or influencer* or "broadcast media" or news* or blog* or internet or digital* or online or on-line or web* or bot or bots or cyber* or "social network*" or "commercial media" or email or clickbait* or "click-bait*" or "mass media").mp. 90997

6 (black* or african* or caribbean or afro* or "person of colo?r" or "people of colo?r" or colo?red or "dark-skin*" or racial* or race? or ((racial or ethnic) adj2 minorit*)).mp. 213815

7 (algeria* or angola* or benin* or Botswana* or "burkina faso" or Burundi or Cameroon or "Cape Verde" or "Central African Republic" or chad or Comoros or Congo* or "cote d'ivoire" or "Ivory Coast" or Djibouti or Egypt* or Guinea* or Eritrea or Ethiopia* or Gabon or Gambia* or ghana* or Kenya* or Lesotho or Liberia* or Libya* or Madagascar or Malawi* or mali* or Mauritania* or Mauritius or Morocco or Mozambique or Namibia* or niger or nigeria* or rwanda* or "sao tome and principe" or senegal* or seychelles or "sierra leone" or somalia* or "south africa" or sudan or swaziland or tanzania* or togo or Tunisia* or uganda* or Zambia* or Zimbabwe*).ti,ab. 329928

8 ("Dominican Republic" or Haiti* or Jamaica* or Barbados or Dominica or Grenada or "Saint Lucia" or trinidad* or Bahamas).mp. 11316

9 ((indigenous or aboriginal* or aborigines or "First Nations" or "first peoples") adj3 australia*).mp. 2731

10 6 or 7 or 8 or 9 505031

11 3 and 4 and 5 and 10 59

EBSCOhost Cumulative Index to Nursing and Allied Health Literature (CINAHL) <1936 - September 27, 2021>
Date of search: September 28, 2021

S1 (((MH "Coronavirus+") OR (MH "Coronavirus Infections+") or (coronavirus* or corona virus* or OC43 or NL63 or 229E or HKU1 or HCoV* or ncov* or covid* or sars-cov* or sarscov* or Sars-coronavirus* or Severe Acute Respiratory Syndrome Coronavirus*)) NOT ((SARS or SARS-CoV or MERS or MERS-CoV or Middle East respiratory syndrome or camel* or dromedar* or equine or coronary or coronal or covidence* or covidien or influenza virus or HIV or bovine or calves or TGEV or feline or porcine or BCoV or PED or PEDV or PDCoV or FIPV or FCoV or SADS-CoV or canine or CCov or zoonotic or avian influenza or H1N1 or H5N1 or H5N6 or IBV or murine corona*)) or (MH "COVID-19") OR (MH "COVID-19 Pandemic") OR (MH "SARS-CoV-2") or(covid or 2019-ncov or ncov19 or ncov-19 or 2019-novel CoV or sars-cov2 or sars-cov-2 or sarscov2 or sarscov-2 or Sars-coronavirus2 or Sars-coronavirus-2 or SARS-like coronavirus* or coronavirus-19 or ((novel or new or nouveau) N2 (CoV or nCoV or coronavirus* or “corona virus” or Pandemi*)) or (variant* N2 (India* or "South Africa*" or UK or English or Brazil* or alpha or beta or delta or gamma or kappa or lambda or "P.1" or "C.37")) or ("B.1.1.7" or "B.1.351" or "B.1.617.1" or "B.1.617.2")) and EM 20190601-20301231

S2 (misinform* or mis-inform* or disinformation or misperception* or misconception* or misinterpret* or "fake news" or infodemic* or info-demic or conspirac* or ((information or fact# or science or scienti* or claim* or knowledge or source* or evidence or statement* or guide*) N4 (false* or mislead* or misled or alternative* or deceptive or deception or poor or bad or wrong or correct* or incorrect or trustworth* or untrustworth* or distrust* or mistrust* or confidence or confident or accurac* or accurate or inaccurate or valid* or invalid* or reliabl* or unreliable)) or rumor* or hoax* or myth* or "urban legend*" or gossip or fallac* or "anti-vaxx*" or "anti-scien*" or "false dichotom*" or pseduoscience or pseudo-science)

S3 (MH "Fake News")

S4 (MH "Pseudoscience") OR (MH "Quackery")

S5 S2 OR S3 OR S4

S6 (MH "Social Media+") OR (MH "Email") OR (MH "Internet Access") OR (MH "World Wide Web")

S7 (MH "Blogs")

S8 (MH "Social Networks") OR (MH "Online Social Networking") OR (MH "Social Networking")

S9 ("social media" or "social site*" or Facebook or twitter or tweet* or instagram* or "tik Tok" or YouTube* or Tumblr or reddit or Qzone or snapchat or "snap chat" or Pinterest or influencer* or "broadcast media" or news* or blog* or internet or digital* or online or on-line or web* or bot or bots or cyber* or "social network*" or "commercial media" or email or clickbait* or "click-bait*" or "mass media")

S10 S6 OR S7 OR S8 OR S9

S11 (MH "Black Persons")

S12 (MH "Africa+") OR (MH "Africa, Northern+") OR (MH "Africa South of the Sahara+") OR (MH "Africa, Eastern+") OR (MH "Africa, Southern+") OR (MH "Africa, Western+")

S13 (MH "West Indies+")

S14 (black* or african* or caribbean or afro* or "person of colo#r" or "people of colo?r" or colo#red or "dark-skin*" or racial* or race# or ((racial or ethnic) N2 minorit*))

S15 (algeria* or angola* or benin* or Botswana* or "burkina faso" or Burundi or Cameroon or "Cape Verde" or "Central African Republic" or chad or Comoros or Congo* or "cote d'ivoire" or

"Ivory Coast" or Djibouti or Egypt* or Guinea* or Eritrea or Ethiopia* or Gabon or Gambia* or ghana* or Kenya* or Lesotho or Liberia* or Libya* or Madagascar or Malawi* or mali* or Mauritania* or Mauritius or Morocco or Mozambique or Namibia* or niger or nigeria* or rwanda* or "sao tome and principe" or senegal* or seychelles or "sierra leone" or somalia* or "south africa" or sudan or swaziland or tanzania* or togo or Tunisia* or uganda* or Zambia* or Zimbabwe*)
S16 ("Dominican Republic" or Haiti* or Jamaica* or Barbados or Dominica or Grenada or "Saint Lucia" or trinidad* or Bahamas)
S17 (MH "First Nations of Australia+")
S18 ((indigenous or aboriginal* or aborigines or "First Nations" or "first peoples") N3 australia*)
S19 S11 OR S12 OR S13 OR S14 OR S15 OR S16 OR S17 OR S18
S20 S1 AND S5 AND S10 AND S19

Results: 113

Scopus via Elsevier <1976 - September 29, 2021>
Date of search: Sept 29, 2021

Search terms Results
(((TITLE-ABS-KEY ((coronavirus* OR "corona virus*" OR oc43 OR nl63 OR 229e OR hku1 OR hcov* OR ncov* OR covid* OR "sars-cov*" OR sarscov* OR "Sars-coronavirus*" OR "Severe Acute Respiratory Syndrome Coronavirus*" OR d614g))) AND NOT ((TITLE-ABS-KEY ((sars OR sars-cov OR mers OR mers-cov OR "Middle East respiratory syndrome or camel*" OR dromedar* OR equine OR coronary OR coronal OR covidence* OR covidien OR influenza AND virus OR hiv OR bovine OR calves OR tgev OR feline OR porcine OR bcov))) OR (TITLE-ABS-KEY ((ped OR pedv OR pdcov OR fipv OR fcov OR sads-cov OR canine OR ccov OR zoonotic OR "avian influenza" OR h1n1 OR h5n1 OR h5n6 OR ibv OR murine AND corona*))))) OR (TITLE-ABS-KEY ((pneumonia OR covid* OR coronavirus* OR corona AND virus* OR ncov* OR 2019-ncov OR sars*) AND wuhan) OR ((2019-ncov OR ncov19 OR ncov-19 OR 2019-novel AND cov OR sars-cov2 OR sars-cov-2 OR sarscov2 OR sarscov-2 OR sars-coronavirus2 OR sars-coronavirus-2 OR "SARS-like coronavirus*" OR coronavirus-19 OR covid19 OR covid-19 OR "covid 2019" OR "B.1.1.7" OR "B.1.351" OR "B.1.617.1" OR "B.1.617.2" OR (variant* W/2 (india* OR "South Africa*" OR uk OR english OR brazil* OR alpha OR beta OR delta OR gamma OR kappa OR lambda OR "P.1" OR "C.37")) OR ((covid OR covid19 OR covid-19) AND pandemic*) OR (coronavirus* AND pneumonia)))) OR (TITLE ((novel OR new OR nouveau) AND (cov OR ncov OR covid OR coronavirus* OR corona AND virus OR pandemi*))) OR (ABS ((novel OR new OR nouveau) AND (cov OR ncov OR covid OR coronavirus* OR corona AND virus OR pandemi*))) OR (KEY ((novel OR new OR nouveau) AND (cov OR ncov OR covid OR coronavirus* OR corona AND virus OR pandemi*))) AND ORIG-LOAD-DATE > 20190630) AND (TITLE-ABS-KEY ((misinform* OR mis-inform* OR

disinformation OR misperception* OR misconception* OR misinterpret* OR "fake news" OR infodemic* OR info-demic OR conspirac* OR ((information OR fact? OR science OR scienti* OR claim* OR knowledge OR source* OR evidence OR statement* OR guide*) W/4 (false* OR mislead* OR misled OR alternative* OR deceptive OR deception OR poor OR bad OR wrong OR correct* OR incorrect OR trustworth* OR untrustworth* OR distrust* OR mistrust* OR confidence OR confident OR accurac* OR accurate OR inaccurate OR valid* OR invalid* OR reliabl* OR unreliable)) OR rumor* OR hoax* OR myth* OR "urban legend*" OR gossip OR fallac* OR "anti-vaxx*" OR "anti-scien*" OR "false dichotom*" OR pseduoscience OR pseudo-science))) AND (TITLE-ABS-KEY ("social media" OR "social site*" OR facebook OR twitter OR tweet* OR instagram* OR "tik Tok" OR youtube* OR tumblr OR reddit OR qzone OR snapchat OR "snap chat" OR pinterest OR influencer* OR "broadcast media" OR news* OR blog* OR internet OR digital* OR online OR on-line OR web* OR bot OR bots OR cyber* OR "social network*" OR "commercial media" OR email OR clickbait* OR "click-bait*" OR "mass media")) AND ((TITLE-ABS-KEY (black* OR african* OR caribbean OR afro* OR "person of colo?r" OR "people of colo?r" OR colo?red OR "dark-skin*" OR racial* OR race? OR ((racial OR ethnic) W/2 minorit*))) OR (TITLE-ABS-KEY (algeria* OR angola* OR benin* OR botswana* OR "burkina faso" OR burundi OR cameroon OR "Cape Verde" OR "Central African Republic" OR chad OR comoros OR congo* OR "cote d'ivoire" OR "Ivory Coast" OR djibouti OR egypt* OR guinea* OR eritrea OR ethiopia* OR gabon OR gambia* OR ghana* OR kenya* OR lesotho OR liberia* OR libya* OR madagascar OR malawi* OR mali* OR mauritania* OR mauritius OR morocco OR mozambique OR namibia* OR niger OR nigeria* OR rwanda* OR "sao tome and principe" OR senegal* OR seychelles OR "sierra leone" OR somalia* OR "south africa" OR sudan OR swaziland OR tanzania* OR togo OR tunisia* OR uganda* OR zambia* OR zimbabwe*)) OR (TITLE-ABS-KEY ("Dominican Republic" OR haiti* OR jamaica* OR barbados OR dominica OR grenada OR "Saint Lucia" OR trinidad* OR bahamas)) OR (TITLE-ABS-KEY ((indigenous OR aboriginal* OR aborigines OR "First Nations" OR "first peoples") W/3 australia*))) AND (LIMIT-TO (DOCTYPE , "ar") OR LIMIT-TO (DOCTYPE , "re"))
Results: 171

Cochrane Library via Wiley <1993 - September 28, 2021>
Date of search: Sept 29, 2021

ID Search Hits
#1 coronavirus* or corona virus* or OC43 or NL63 or 229E or HKU1 or HCoV* or ncov* or covid* or sars-cov* or sarscov* or Sars-coronavirus* or Severe Acute Respiratory Syndrome Coronavirus* 8458
#2 SARS or SARS-CoV or MERS or MERS-CoV or Middle East respiratory syndrome or camel* or dromedar* or equine or coronary or coronal or covidence* or covidien or influenza virus or HIV or bovine or calves or TGEV or feline or porcine or BCoV or PED or PEDV or

PDCoV or FIPV or FCoV or SADS-CoV or canine or CCov or zoonotic or avian influenza or H1N1 or H5N1 or H5N6 or IBV or murine corona* 107863

#3 #1 NOT #2 4227

#4 [mh "Covid-19"] 583

#5 covid or covid19 or ((novel or new or nouveau) NEAR/2 (CoV or nCoV or covid or coronavirus* or corona virus or Pandemi*)) or (variant* NEAR/2 (India* or "South Africa" or UK or English or Brazil* or alpha or beta or delta or gamma or kappa or lambda or "P.1" or "C.37")) or "B.1.1.7" or "B.1.351" or "B.1.617.1" or "B.1.617.2" 7354

#6 #3 or #4 or #5 7495

#7 (misinform* or mis-inform* or disinformation or misperception* or misconception* or misinterpret* or "fake news" or infodemic* or info-demic or conspirac* or ((information or fact? or science or scienti* or claim* or knowledge or source* or evidence or statement* or guide*) NEAR/4 (false* or mislead* or misled or alternative* or deceptive or deception or poor or bad or wrong or correct* or incorrect or trustworth* or untrustworth* or distrust* or mistrust* or confidence or confident or accurac* or accurate or inaccurate or valid* or invalid* or reliabl* or unreliable)) or rumor* or hoax* or myth* or "urban legend*" or gossip or fallac* or "anti-vaxx*" or "anti-scien*" or "false dichotom*" or pseduoscience or pseudo-science) 16899

#8 ("social media" or "social site*" or Facebook or twitter or tweet* or instagram* or "tik Tok" or YouTube* or Tumblr or reddit or Qzone or snapchat or "snap chat" or Pinterest or influencer* or "broadcast media" or news* or blog* or internet or digital* or online or on-line or web* or bot or bots or cyber* or "social network*" or "commercial media" or email or clickbait* or "click-bait*" or "mass media") 1819496

#9 (black* or african* or caribbean or afro* or "person of colo?r" or "people of colo?r" or colo?red or "dark-skin*" or racial* or race? or ((racial or ethnic) NEAR/2 minorit*)) 41166

#10 (algeria* or angola* or benin* or Botswana* or "burkina faso" or Burundi or Cameroon or "Cape Verde" or "Central African Republic" or chad or Comoros or Congo* or "cote d'ivoire" or "Ivory Coast" or Djibouti or Egypt* or Guinea* or Eritrea or Ethiopia* or Gabon or Gambia* or ghana* or Kenya* or Lesotho or Liberia* or Libya* or Madagascar or Malawi* or mali* or Mauritania* or Mauritius or Morocco or Mozambique or Namibia* or niger or nigeria* or rwanda* or "sao tome and principe" or senegal* or seychelles or "sierra leone" or somalia* or "south africa" or sudan or swaziland or tanzania* or togo or Tunisia* or uganda* or Zambia* or Zimbabwe*) 66738

#11 ("Dominican Republic" or Haiti* or Jamaica* or Barbados or Dominica or Grenada or "Saint Lucia" or trinidad* or Bahamas) 1161

#12 ((indigenous or aboriginal* or aborigines or "First Nations" or "first peoples") NEAR/3 australia*) 279

#13 #9 or #10 or #11 or #12 102856

#14 #6 and #7 and #8 and #13

Results: 62 Reviews, 15 Trials
